# Haloperidol-induced facial and upper limbs oedema: a case report study

**DOI:** 10.1192/j.eurpsy.2023.2118

**Published:** 2023-07-19

**Authors:** S. Khayeche, B. Saguem, R. Majdoub, J. Nakhli

**Affiliations:** Psychiatry, Farhat Hached hospital, Sousse, Tunisia

## Abstract

**Introduction:**

Haloperidol is one of the first-generation antipsychotics which is used widely around the world and still has its place in psychotic disorders along with his documented antimanic properties.

It acts by blocking essentially dopaminergic receptors but also serotonergic and alpha-adrenergic receptors.

Haloperidol has many side effects, especially extrapyramidal symptoms. Oedema associated with haloperidol is a rare side effect.

**Objectives:**

The aim of this presentation is to describe the case of a young female who developed a rare side effect of haloperidol.

**Methods:**

we conducted a review of literature on different data base about this side effect in order to discuss its potential underlying mechanisms.

**Results:**

Here we report a case of a 24-year-old female with the history of bipolar disorder type 1, and an allergy to chlorpromazine. She was admitted to our psychiatric department for a manic episode with psychotic features and was initially treated with 50 mg of Haloperidol in addition to 30 mg of valium per day. She developed pronounced oedema of the face and upper limbs after two weeks of treatment. All of the paraclinical examinations including blood cell count, liver function tests, renal function tests, serum electrolytes, ECG, urine test didn’t show any abnormalities. We solicited the opinion of both nephrology and pharmacovigilance and concluded to an allergic oedema.

The symptoms disappeared 10 days after the discontinuation of haloperidol which suggested a potential incrimination of this drug

As far as we know, there are very few reports of allergic oedema with haloperidol in the literature. Potential underlying mechanisms will be discussed.

**Conclusions:**

Through this case study, we aimed to focus attention on this very rare but still possible side effect.

**Disclosure of Interest:**

None Declared

